# Bronchial anastomotic complications as a microvascular disruption in a mouse model of airway transplantation

**DOI:** 10.3389/fimmu.2025.1567657

**Published:** 2025-05-14

**Authors:** Mohammad Afzal Khan, Subarna Bhusal, Christine L. Lau, Alexander Sasha Krupnick

**Affiliations:** Department of Surgery, University of Maryland, Baltimore, MA, United States

**Keywords:** lung transplant - ischemia-reperfusion injury, microvascular abnormalities, airway anastomosis, airway anastomotic complications, tissue repair and organ regeneration

## Abstract

Lung transplantation (LTx) offers a last resort for patients battling end-stage lung disease. Even though short-term survival has improved, these patients still face several long-term challenges, such as chronic rejection and ischemic bronchial anastomosis. In lung transplant recipients, the bronchial anastomosis is prone to complications—such as poor wound healing, necrosis, stenosis, and dehiscence—due to the marginal blood supply at this site. During peri-LTx, hypoxia and ischemia stimulate fibrotic and inflammatory cytokines at anastomotic sites, leading to abnormal collagen production and excessive granulation, which impair wound healing. Despite meticulous techniques, bronchial anastomosis remains a major cause of morbidity and mortality among lung transplant recipients. After LTx, most bronchial complications are attributed to ischemic insult since normal bronchial blood flow is disrupted, and bronchial revascularization usually takes two to four weeks, making the anastomotic bronchial vessels dependent on pulmonary artery circulation. It is clear that hypoxia, inflammation, oxidative stress, and extracellular matrix remodeling play critical roles in bronchial complications, but there is no small animal model to study them. In the context of LTx, mouse tracheal models are essential tools for studying bronchial complications, particularly ischemia, fibrosis, and stenosis, as well as evaluating potential therapeutic interventions. A well-established mouse model of orthotopic tracheal transplantation (OTT) mimics the anastomosis of the bronchi and the subsequent microvascular injury, providing a pathological correlation with anastomotic complications. A series of previous studies using the OTT model explored the microvascularization, ischemia-reperfusion, airway epithelial injury, and fibrotic remodeling effects after airway anastomosis. This review describes OTT as a model of airway anastomotic complications, which is crucial for understanding the immunological and molecular pathways as seen in clinical bronchial anastomoses, as well as improving anastomotic healing and reducing complications through targeted therapeutic strategies.

## Introduction

**Figure 1:**
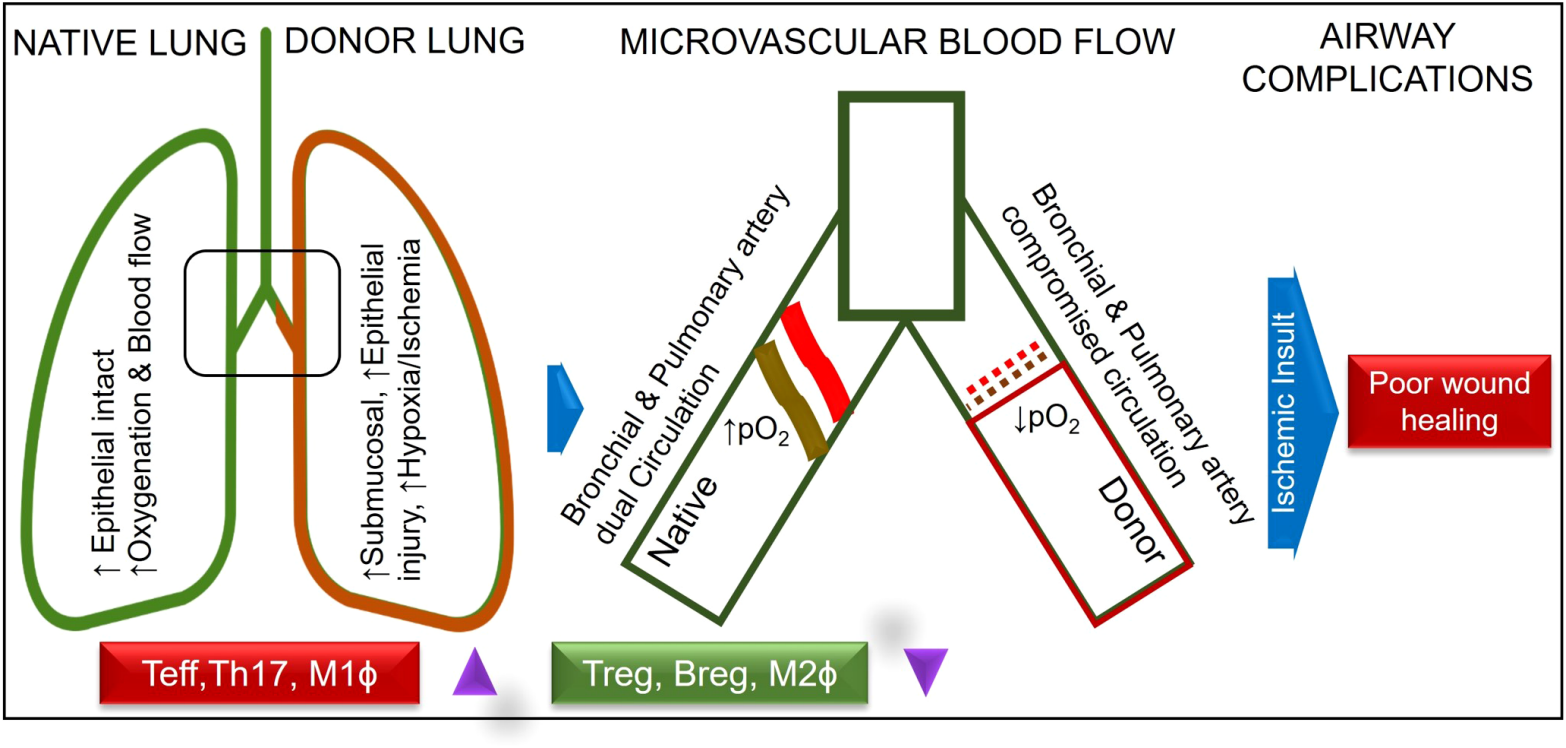
Normal and compromised dual circulation in native and donor lung post lung-transplantation.

Lung graft rejection commonly presents as acute cellular rejection with symptoms like shortness of breath, cough, fever, or asymptomatic pulmonary functions test (PFT) decline; chronic rejection such as bronchiolitis obliterans syndrome (BOS) leads to irreversible airflow obstruction and progressive respiratory decline; and antibody-mediated rejection involves donor-specific antibodies, capillaritis, and respiratory distress—all contributing to increased infection risk, reduced graft function, and complications from immunosuppression (1). Bronchial anastomotic complications after lung transplantation (LTx), can significantly impact graft function and patient outcomes, necessitating prompt diagnosis and intervention (2). The bronchial anastomosis has been clinically recognized as the most vulnerable site for operative complications and is defined as a lack of vascular perfusion, poor wound healing, stenosis, necrosis, and dehiscence of the bronchus of the donor lung and recipient (3–10). At bronchial anastomotic sites, hypoxia and ischemia stimulate profibrotic and inflammatory cytokines, impairing wound healing through abnormal collagen production and excessive granulation. During anastomosis, the recipient’s main bronchi are supplied by native bronchial arteries, while the donor’s bronchi are supplied by collateral circulation, and the revascularization of the donor by the recipient may take up to four weeks, which may leave the airways vulnerable to ischemic damage (9). Bronchial anastomotic complications after LTx can be caused by a number of factors, including bronchial ischemia, infection, immunosuppression, surgical technique, donor and recipient factors and organ preservation (11). It is believed that ischemic insults are the most common cause of airway complications because normal blood flow supply is interrupted, leaving the bronchial vessels at the anastomotic site dependent on arterial circulation following lung transplantation (9, 12). As a result of hypoxia and ischemia, major inflammatory and profibrotic cytokines are released, causing an imbalance in collagen synthesis, degradation, and excessive proliferation of granulation tissue (6, 13–15). Bronchial anastomotic complications are a major complication that can contribute to morbidity and mortality, and occur within the first year of transplantation (16). The incidence of lethal and nonlethal airway complications has decreased since the early experiences; however, it results in considerable morbidity, decreased quality of life, increased cost, and account for a mortality of 2% - 4% (3, 3, 16–20). Bronchial anastomotic complications can be treated with a multispecialty team approach, including medical management and interventional bronchoscopic procedures (21). A variety of surgical techniques have been used to overcome complications associated with bronchial anastomoses (22, 23). Despite advances in lung transplantation, airway complications after LTx remain unchanged in terms of their risk factors, incidence, and lethality. Complications related to airway grafts, an infected environment, and prolonged mechanical ventilation remain obstacles (4, 19, 24, 25). Although only a limited number of therapeutic options have been used to promote bronchial anastomotic healing, especially platelet rich plasma or platelet-derived growth factor (PDGF) therapy, their effectiveness has been limited (26, 27). The occurrence of bronchial anastomotic complications is associated with a number of factors, including primary graft dysfunction, acute rejection (AR), preoperative and postoperative pulmonary infections, prolonged mechanical ventilation, single lung transplantation, and Aspergillus fumigatus colonization (28). AR is associated with severe inflammation through perivascular, subendothelial and mononuclear infiltrates, submucosal edema, reduced graft perfusion, and poor wound healing, which are crucial risk factors for airway complications (4, 12, 19, 20, 22, 27, 29). The delay or reversal of AR with immunotherapy is highly dependent on the presence of a functional microcirculation in the graft (30). Therefore, therapies that specifically preserve the microcirculation could prevent allograft rejection and potentially subsequent fibrotic remodeling. Studies have shown that alloimmune inflammation adversely affects the microvascular connections between recipients and donors of rejecting allografts compared with non-rejecting syngrafts (31–34) (**Table 1**; **Figure 1**).

**Table 1:** Most common pathological changes associated with lung transplantation.

Type of Rejection	Time-frame	Associated issues
Acute rejection	Usually within the first year	Inflammation of the lung tissue, Decline in lung function, Bronchiolitis
Chronic rejection	often starts after the first year	Bronciolitis Obliterans Syndrome (BOS), Restrictive Allograft Syndrome (RAS), Airway complications, Recurrent or persistent infections, Alloimmune injury from HLA antibodies

### Bronchial anastomosis can be replicated in small animals as a microvascular disruption between donor to recipient airway grafts

**Figure 2:**
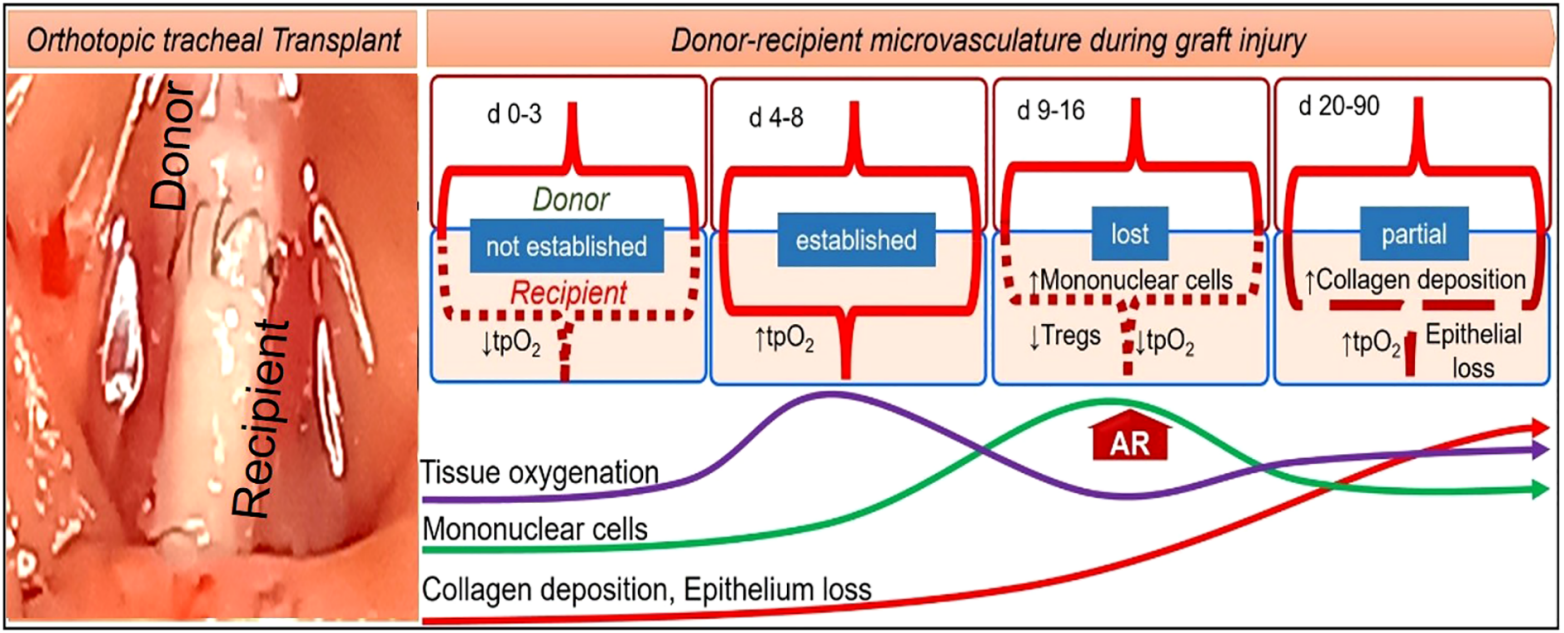
A mouse model of orthotopic tracheal transplantation, which demonstrates donor-recipient microvascular connections, tissue oxygenation, inflammation, collagen, and epithelial loss post-tracheal transplantation.

A severe hypoxic state and impaired blood flow due to massive intragraft infiltration of inflammatory cells are believed to be the primary causes of poor wound healing and associated complications in the bronchus after LTx (5, 10, 32, 35–38). Orthotopic tracheal transplantation (OTT) in mice is a well-established model that mimics bronchial anastomosis and subsequent microvascular injury post-tracheal transplantation (39). It has been demonstrated in OTT model that microvascular supplies between donors and recipients begin at day (d) 4, remain connected until day 8, and start disappearing by day 10 of tracheal transplantation in rejecting allografts, which coincides with a decline in tissue oxygenation and microvascular blood flow as compared to the non-rejecting syngrafts that are constantly oxygenated (31, 32). Additionally, pathological examinations revealed that rejecting allografts showed denuded graft epithelium during acute rejection followed by this massive deposition of subepithelial collagen and airway narrowing post-transplantation. There has been clinical evidence that adequate microvascular supply and oxygen saturation in the airway tissue are vital for lung transplantation, which was later validated in preclinical OTT studies (5, 6, 39, 40). The involvement of transforming growth factor-beta (TGF-β) and inflammatory cytokines such as interleukin-6 (IL-6) and tumor necrosis factor-alpha (TNF-α) mirrors the molecular changes seen in human patients (41). In addition, a variety of animal and *in-vitro* models have been developed for examining lung transplant complications, though each has some limitations (42). In particular, mouse trachea transplant models are widely used to study airway stenosis, replicating the pathological conditions observed in human patients with post-surgical airway narrowing, fibrosis, and inflammation (39, 42, 43). These preclinical studies exhibit key pathological features of clinical airway stenosis, including epithelial denudation, fibroblast proliferation, extracellular matrix remodeling, and excessive collagen deposition (42, 44). These tracheal models are essential for testing anti-fibrotic drugs, gene therapies, and regenerative approaches like stem cell therapy. In addition, these models also allow for the investigation of various bronchial complication, providing valuable insights into potential therapeutic interventions for various pathological issues. Their small size, genetic manipulability, and cost-effectiveness make them a powerful tool for studying airway stenosis and developing novel treatments to improve airway healing and function (**Figure 2**).

## Immune response during airway anastomosis

**Figure 3:**
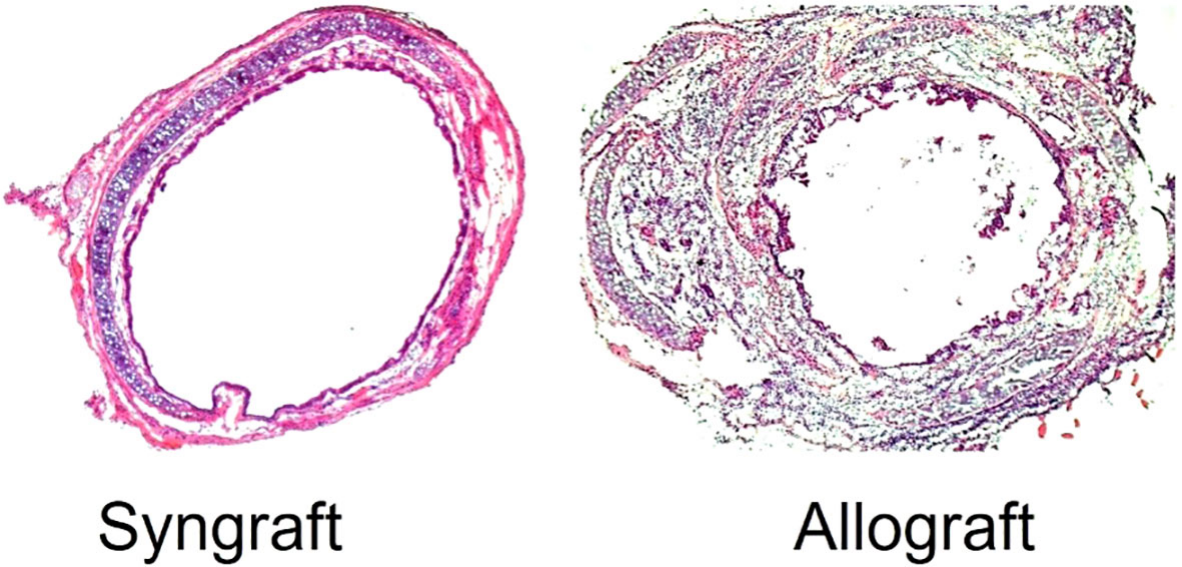
A mouse airway transplant shows an airway narrowing during alloimmune inflammation.

CD4^+^ T cells play an important role during alloimmune inflammation, while Tregs and associated mediators regulate immune responses to prevent ongoing tissue damage (33, 36, 37, 45–52). In organ transplantation, inflammatory cytokines such as TNF-α, IL-2, IL-1β, and IL-6 also contribute to the immune-mediated destruction of the graft’s airway epithelium, vascular endothelium, and cilia during wound healing (53, 54). Inflammatory cytokines such as TNF-α and interleukins (IL-6, IL-8) contribute to local tissue damage and fibrosis, exacerbating bronchial stenosis (55), however, oxidative stress, driven by reactive oxygen species (ROS), further damages cellular components, impairing healing processes (56).In contrast, Tregs, M2 macrophages, and regulatory mediator s (IL-33, TGF-β, PDGF, and TSG-6) promote the repair of both airway epithelium and vascular endothelium (57–67). As a consequence of alloimmune inflammation (T cells, B cells, macrophages, active complement factors, and immunoglobulins), microvascular loss and associated tissue injuries lead to tissue remodeling, the closure of small airways, and the subsequent decline of respiratory function (5, 6, 30, 40, 68). There is, however, an association between inflammation-induced microvascular loss and airway epithelial injury, which is a major pathological reason for graft malfunction, airway narrowing and subepithelial fibrosis in allograft rejection (30). Consequently, based on the previous research therapies targeting various regulatory cells/mediators could control alloimmune inflammation, thereby promoting graft microvascular repair, airway narrowing and suppressing fibrosis (31, 69–72) (**Figure 3**).

Preclinical studies in OTT model have shown significant increases in CD4^+^ and CD8^+^ T cells in peripheral blood and tracheal graft from allografts compared to syngrafts (32, 35). In addition, syngrafts showed significantly elevated levels of FOXP3^+^ Tregs and IL-10 as compared to rejecting allografts. These findings indicated that functional microvascular loss during rejection is associated with the upregulation of both peripheral and graft infiltration of CD4^+^, CD8^+^ T cells, IL-2, TNF-α and STAT3 while a downregulation of FOXP3^+^ Tregs and IL-10 post-transplantation (60, 61, 73, 74). In addition, studies in OTT and other transplantation have demonstrated that alloimmune inflammation augments increase in inflammatory cell phenotypes and associated mediators, while downregulated FOXP3^+^ Tregs, and IL-10 in the graft, which triggers progression of airway epithelial injuries, and thus wound healing after transplantation (32, 35, 74). Various therapeutic approaches have been adapted preserve microvasculature through different regulatory mediators including regulatory cells/stem cells, IL-10, C5a blockade and CTLA-4Ig blockade in tracheal transplantation (44, 60, 61, 75). In a therapeutic approach with mesenchymal stem cells-mediated (MSCs) or regulatory T cells adoptive transfer, we successfully restored immunetolerance, through the upregulation of FOXP3^+^ Tregs, TSG-6 and IL-10 in the grafts (75–81). While C5a blocking significantly leads to enhanced presence of Tregs in the allograft, reinstates donor–recipient functional microvasculature, improves tissue oxygenation, microvascular blood flow, and epithelial repair, followed by an upregulation of IL-5, TGF-β, IL-10 vascular endothelial growth factor, and ANGPT1 gene expression, while it maintained a healthy epithelium and prevented subepithelial collagen deposition after 4-weeks of airway transplantation (35, 37, 51, 73). Together, C5a signaling has potential to preserve microvasculature and rescue allograft from a sustained hypoxic/ischemic phase, limits airway tissue remodeling through the induction of Treg-mediated immune tolerance. In another approach, CTLA4-Ig mediated immunosuppression significantly resulted in late increases in both peripheral CD4^+^/CD8^+^ FOXP3^+^ Tregs and serum IL-10, but prevents the microvascular deposition of IgG, complement factor C3d, and epithelial C4d respectively, which proportionally improved blood flow and tissue oxygenation in the graft and, thus, promotes graft repair. Also, it restored the airway lumen, epithelium, and prevented the progression of subepithelial collagen deposition up to 90 days after transplantation. This study demonstrated that CTLA4-Ig-mediated immunosuppression potentially modulates both effector response and a late surge of regulatory activity to preserve graft microvasculature and rescue allograft from sustained hypoxia and ischemia and thereby limits subepithelial fibrosis (44). All these therapeutic approaches highlighted that IL-10 mediated immunosuppression established the immune tolerance phase and thereby modulated both microvascular and epithelial integrity, which affected inflammation-associated graft malfunctioning and sub-epithelial fibrosis in rejecting allografts. Further studies investigated the reparative effects of IL-10 on microvasculature and epithelium in a mouse model of airway transplantation. To investigate the IL-10 mediated microvascular and epithelial repair, we depleted and reconstituted IL-10, and monitored graft microvasculature, airway epithelium, and associated repair proteins. Our data demonstrated that both untreated control allografts and IL-10 (−) allografts showed a significant early increase in microvascular leakiness, drop-in tissue oxygenation, blood perfusion, and denuded airway epithelium, which is associated with loss of adhesion protein Fascin-1 and β-catenin on vascular endothelial cells post-transplantation. However, IL-10 (+) promotes early microvascular and airway epithelial repair, and a proportional increase in endothelial Fascin-1, and β-catenin post-transplantation. Moreover, airway epithelial cells also express a significantly higher expression of FOXJ1 and β-catenin in syngrafts and IL-10 (+) allografts as compared to IL-10 (−) and untreated controls post-transplantation. Collectively, these findings demonstrated that IL-10 mediated microvascular and epithelial changes are associated with the expression of FOXJ1, β-catenin, and Fascin-1 proteins on the airway epithelial and vascular endothelial cells, respectively. These findings established a potential reparative modulation of IL-10 associated microvascular and epithelial repair, which could provide a vital therapeutic strategy to facilitate bronchial anastomotic graft repair in clinical settings (13, 44, 60, 61, 73–75).

## Hypoxia and ischemia

In lung transplantation, hypoxia and ischemia during bronchial anastomosis are critical concerns that can significantly impact the success of the procedure (10, 82). Both hypoxia and ischemia result in impaired cellular function and reduced wound healing, which can lead to complications like anastomotic dehiscence, infection, and scarring (6, 83, 84). The immune response triggers the release of inflammatory mediators that further impair vascular function and increase the risk of ischemic damage (6, 10, 83, 85, 86). These conditions can impact the healing of the bronchial anastomosis, leading to complications such as anastomotic leak, airway stenosis, or graft dysfunction (16). Hypoxia-inducible factor-1 alpha (HIF-1α) plays a key role in cellular adaptation to hypoxic conditions at the anastomotic site (87). Under oxygen deprivation, HIF-1α activation promotes angiogenesis via vascular endothelial growth factor (VEGF), but excessive hypoxia can lead to inadequate revascularization, increasing the risk of ischemia-related complications (88). Chronic ischemia can result in fibrosis and scarring of the bronchial walls, leading to bronchial stenosis, which can cause airflow obstruction (89). Ischemia refers to a reduced blood supply to the grafted lung tissue, which can occur during the surgical procedure, especially when there is tension at the bronchial anastomosis or if the vascular supply is compromised (6). Ischemia can also occur postoperatively if there is inadequate perfusion, leading to necrosis of the bronchial tissues (90). In addition, ischemia can lead to delayed healing and increase the risk of airway complications such as stenosis or bronchomalacia, as the prolonged lack of oxygen and nutrients to the bronchial tissues impair the cellular repair processes, causing fibrosis or weakening of the anastomosis (11). The mechanisms of hypoxia and ischemia during bronchial anastomosis after LTx are closely tied to the disruption of normal blood flow to the bronchial tissues and the unique challenges faced during this delicate surgical procedure (5). When performing the bronchial anastomosis, it’s critical that blood flow to the bronchial tissue is maintained or restored, and If there is poor vascularization, it leads to insufficient oxygen delivery to the anastomotic site, which results in hypoxia (10). The donor lung’s bronchial arteries are often different from those of the recipient, and in many cases, these donor vessels might not have robust anastomotic branches with the recipient’s vascular system, leading to poor perfusion (19). During the bronchial anastomoses, the bronchus may undergo handling that causes damage to microvessels in the area around the anastomosis, which disrupts the local blood flow, contributing to ischemia and hypoxia (7, 30). Furthermore, tension at the anastomotic site, such as when the bronchial tubes are not aligned properly or when excessive tension is placed on the sutures, can lead to vessel constriction or occlusion, further impeding blood flow (9). After the anastomosis, oxygen must diffuse from the surrounding tissues to the bronchial walls, and in the absence of an optimal blood supply, this diffusion is impaired, which leads to hypoxic conditions in the bronchial tissue (9, 91). Ischemia occurs when there is insufficient blood supply to a tissue, leading to a shortage of oxygen and nutrients required for cell survival and tissue function (15, 83). Several mechanisms contribute to ischemia during bronchial anastomosis, and one of the primary causes of ischemia after lung transplantation is the interruption of normal blood flow when the donor lung is surgically connected to the recipient’s vasculature (10, 82). If the bronchial arteries are not adequately reconnected or if the blood vessels are not aligned properly, the blood flow to the bronchus can be limited or obstructed (6, 92). In some cases, especially in older transplant techniques, the bronchial arteries of the donor may be ligated or not re-anastomosed, relying on collateral circulation, which may not be sufficient for proper oxygenation of the bronchial tissues (93). During the dissection and implantation process, there is a risk of damaging the small vessels that supply the bronchial tree, and if these vessels are compromised, blood flow can be reduced or completely interrupted, leading to ischemic injury (9). The endothelial lining of the blood vessels can also become dysfunctional due to ischemic injury, further impairing the ability of vessels to dilate and increase blood flow to the area (86). There are factors that contribute to both Hypoxia and Ischemia which include Inadequate anastomotic techniques and infection (94). Infection or inflammation at the site of the bronchial anastomosis can lead to local vasculitis, which can further compromise blood supply and exacerbate ischemic damage (95). A poorly executed bronchial anastomosis may not only lead to hypoxia but also contribute to ischemia if the blood vessels are pinched, compressed, or disconnected inappropriately during suturing, which involves carefully placing stitches that minimize tension and avoid damaging the vasculature (95). Rejection responses in the early post-transplant period can lead to vascular changes (e.g., endothelial injury, vasculitis), reducing blood flow to the bronchial tissues and contributing to ischemia and hypoxia. Ischemia is usually monitored using advanced imaging and monitoring techniques during surgery to ensure that the blood flow to the transplanted bronchus is adequate, which may include the use of Doppler ultrasound or other intraoperative assessments to evaluate vascular patency (89). In addition, careful attention to suturing techniques to minimize tension on the bronchus and to ensure proper alignment and blood supply to the anastomosis, ensuring an adequate vascular pedicle for the transplanted lung can help reduce ischemia. Early detection of complications through close monitoring of the patient’s respiratory status, chest imaging, and bronchoscopy to assess the integrity of the bronchial anastomosis and identify any signs of hypoxia, ischemia, or airway obstruction (96, 97). Regular follow-up with imaging and pulmonary function tests is critical to monitor for these complications, however, even after the anastomosis heals, chronic ischemia or hypoxia could result in long-term issues such as airway stenosis or bronchiectasis, which can impact the patient’s lung function over time.

**Figure 4:**
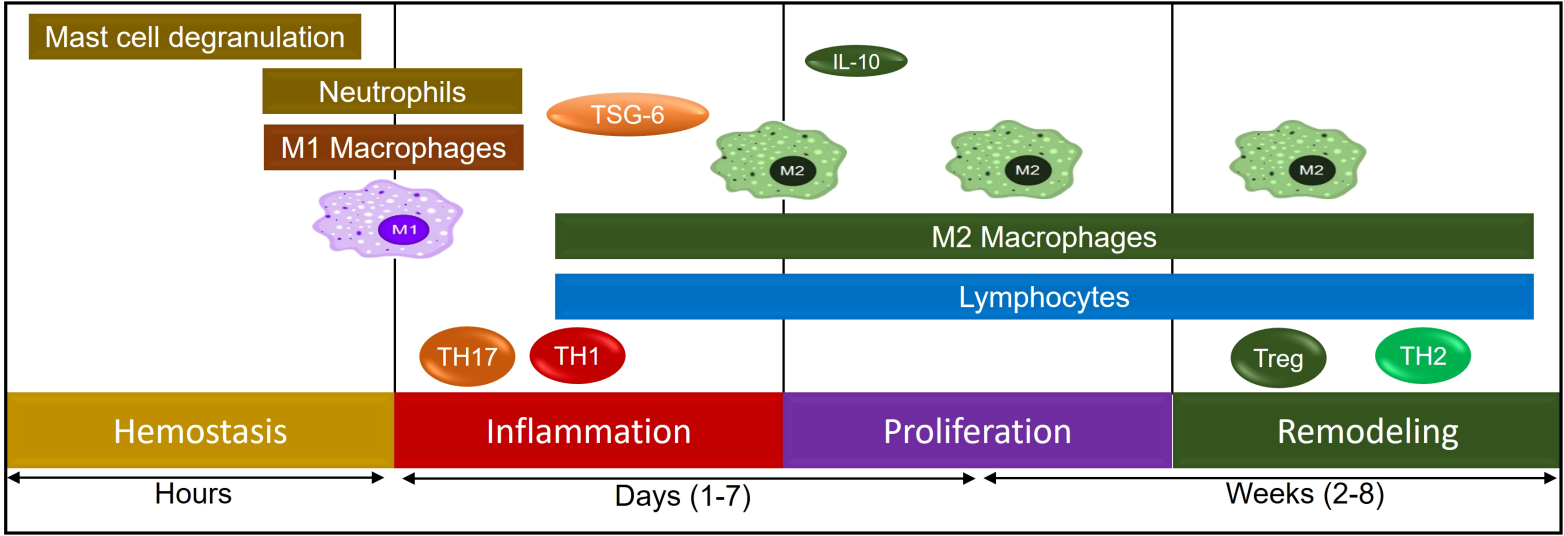
A schematic representation of various immune cells during the process of tissue remodeling.

Hypoxia triggers several keys signaling pathways in cells to adapt to the reduced oxygen supply, and these pathways are critical for cellular survival, angiogenesis and metabolic adaptation. The main signaling pathway triggered by hypoxia is the Hypoxia-Inducible Factor (HIF) pathway, though other pathways also play important roles in mediating cellular responses to hypoxia which include AMP-Activated Protein Kinase (AMPK) Pathway, Mitogen-Activated Protein Kinase (MAPK) Pathway, Nitric Oxide (NO) Pathway, Toll-Like Receptor (TLR) Pathway, Sirtuin Pathway, Hypoxia-Activated Signaling through the PI3K/Akt Pathway, and JAK/STAT Pathway (13, 98–100). The HIF pathway is the central molecular response to hypoxia, and it involves a family of transcription factors, primarily HIF-1 and HIF-2, which regulate the expression of genes involved in adaptation to low oxygen levels (101). In normoxic conditions, HIF-1α (a subunit of HIF-1) is hydroxylated by prolyl hydroxylase enzymes in the presence of oxygen, and this hydroxylation marks HIF-1α for degradation by the proteasome. HIF-1β (the other subunit of HIF-1) remains stable and forms a dimer with HIF-1α when it is available. However, without the accumulation of HIF-1α, the complex does not translocate to the nucleus. Under hypoxic conditions, the activity of prolyl hydroxylases is inhibited due to the lack of oxygen, preventing the hydroxylation and degradation of HIF-1α. As a result, HIF-1α accumulates, translocate to the nucleus, and dimerizes with HIF-1β. The HIF complex then binds to specific DNA sequences (hypoxia-responsive elements or HREs) in the promoters of target genes. These genes take active part in various process like angiogenesis where it promotes the expression of genes like VEGF (vascular endothelial growth factor), which stimulates the formation of new blood vessels to improve oxygen delivery. HIF also activates genes involved in anaerobic metabolism, such as GLUT1 (glucose transporter 1) and LDHA (lactate dehydrogenase), which enhance glucose uptake and lactic acid production, allowing the cell to produce energy in the absence of oxygen (102). HIF stimulates the production of erythropoietin (EPO), which promotes red blood cell production to increase oxygen-carrying capacity (103, 104) (**Figure 4**).

## Bronchial fibrosis and stenosis

The phase of hypoxia and ischemia peri lung transplantation LTx stimulates the production of inflammatory and profibrotic cytokines, leading to an imbalance in collagen synthesis and excessive proliferation of granulation tissue, which contributes to slow wound healing and multiple anastomotic issues that impair patient outcome (10, 90, 105). Clinical studies confirm that changes in airway oxygenation and perfusion during the development and resolution of airway obstructions are relatively understudied aspects of pulmonary physiology (5, 39, 89, 106). There has been evidence in clinical studies that ischemic injury is manifested by endothelial dysfunction resulting in increased vascular permeability and proinflammatory cytokines, which negatively affect transplantation outcomes (15). Bronchial fibrosis and bronchial stenosis are both conditions that can affect the bronchial tubes, which are muscular structures that branch off the trachea into the lungs (16). Bronchial fibrosis can cause bronchial stenosis due to external luminal constriction, which is an abnormal narrowing of the bronchial tubes that can be caused by a number of factors, including. Fibrosis is a type of bronchial anastomotic complication that can occur after lung transplantation, which can result in shortness of breath, bronchial stenosis, and anastomosis dehiscence. Airway fibrosis after lung transplantation can occur due to a number of factors, including bronchial artery injury, poor graft preservation, debridement of the peri-bronchial tissue during anastomosis can increase ischemic injury. The molecular events that contribute to fibrosis and stenosis include Cell proliferation, Inflammation, ECM degradation, MicroRNA expression, Connective tissue stiffening, and Immune responses (107). Various proinflammatory cytokines have been associated with fibrosis, which include IL-4, IL-13, IL-1, TGF-b1, IL-17-A, while IL-10 has been widely recognized as antifibrotic (108). The relationship between inflammation and fibrogenesis has led to IL-10 being identified as a potential antifibrotic target as well as a gatekeeper of fibrotic/antifibrotic signaling, so immune and cell-based therapies aiming to capitalize on IL-10 as a target could be effective in treating lung transplanted patients suffering from delayed would healing. The mechanisms that cause airway fibrosis are complex and not fully understood. However, some of the factors that may be involved include epithelial and endothelial injury, Inflammation, Oxidative stress, TGF-β, reduced autophagy in lung epithelial cells and fibroblasts can aggravate inflammation and fibrosis (109). TGF-β: This is a profibrotic mediator that can promote epithelial-mesenchymal transition (EMT), fibroblast activation. During fibrosis, activated fibroblasts synergize with TGF-β1 to promote extracellular matrix and collagen deposition (109). Matrix metalloproteinases (MMPs) are endopeptidases that contribute to the restoration of tissue architecture by eliminating damaged components of the extracellular matrix (ECM). MMP activation in the setting of a stable airway is due to alloantigen presentation, and a direct contact between T lymphocytes and monocytes has been described as a major pathway for MMP expression (110). Activated T cells induced MMP-9 from macrophages and fibroblasts, elevated levels of MMP, particularly MMP-8, MMP-9 and TIMP-1 were found in BAL fluid in transplant patients (111). Endothelial cells are critical in synthesizing growth factors such as vascular endothelial growth factor (VEGF), platelet derived growth factor (PDGF), TGFβ1 and endothelin-1(ET-1) (112). Instances where microvascular lumen is occluded by a thick collagen layer, growth factor pathways appear to be dysregulated, and this pathway therefore further adds to the scar burden in the development of bronchial stenosis (11). This pathological alteration is a common airway complication after lung transplantation that can cause a number of problems, including dyspnea, cough, wheezing, declining flow rates on spirometry, and recurring post-obstructive pneumonia (11, 19, 113). Occurrence of fibrosis a crucial factor during bronchial anastomotic complications, and extent has been extensively studied in mouse model of orthotopic tracheal transplantation, which replicated bronchial anastomoses. In tracheal transplantation model, it has been shown that progression of subepithelial collagen deposition starts soon after graft microvascular disruption, which further leads to tracheal narrowing (32, 35, 44, 60, 61, 74, 75).

Anastomotic stenosis occurs at the surgical site due to insufficient blood supply to the donor-tracheobronchial tree, which can lead to ischemia, anastomotic dehiscence, malacia and stenosis (114, 115). Bronchial stenoses are challenging complications after lung transplantation and are associated with high rates of morbidity and mortality (113, 114). While in clinical lung transplantation, airway stenosis and fibrosis are common complications after lung transplantation and can significantly impact a patient’s long-term prognosis. Studies have reported that up to 40% of lung transplant patients experience perianastomotic stenosis, and up to 4% experience non-anastomotic distal bronchial stenoses. Angiogenesis is another mechanism which can add insult to already ischemic bronchus. In murine orthotopic trachea transplant (OTT) models repair of airway microvasculature is repaired by recipient derived precursor cells via HIF-1α pathway. HIF-1α expression was also linked to microvascular integrity and *Aspergillus fumigatus* host-pathogen interaction in murine OTT model. Angiogenesis and extracellular matrix (ECM) deposition increases at the donor-recipient interface via the usual sequential phases of hemostasis via fibrin clot, inflammatory phase of neutrophil mediated microbe/foreign body destruction, proliferative phase of granulation tissue deposition and finally the maturation phase of tissue remodeling. After lung transplant, re-establishment of this capillary network is done predominantly by recipient derived Tie2^+^ (tunica intima endothelial tyrosine kinase 2) positive endothelial cells. This capillary restoration is complicated by the fact that microvasculature of airways subjected to alloimmune rejection and are unstable with small caliber arterioles, venules, have relatively poor pericyte coverage and are less efficient in delivery of nutrients and oxygen to injured airways. Angiogenesis is either driven by hypoxia (8) or occurs downstream to deposition of fibrin clot and subsequent inflammatory secretion of growth factors such as (be more specific) vascular endothelial growth factor (VEGF), fibroblast growth factor (FGF) and transforming growth factor beta (TGF-β). Hypoxia mediated cellular response is driven primarily by the hypoxia-inducible-factor (HIF) pathway. Since HIF-dependent changes in metabolism affect the phenotype and function of immune cells, the two pathways leading to angiogenesis i.e. hypoxia and inflammation are interconnected. In addition, failure of angiogenesis at the donor-recipient interface and resultant necrosis leads to 1-10% reported incidences of dehiscence (116). Another airway complications, bronchomalacia, is caused by atrophy of longitudinal elastic fibers and replacement of cartilaginous bronchial rings by fibrous collagen (24). Diffuse bronchomalacia involving the entire bronchus also has been described in the era of tracheal anastomosis but no case reports of diffuse bronchomalacia were found in the current literature that involves sequential double lung transplant with bronchial anastomosis.

## Wound healing

**Figure 5:**
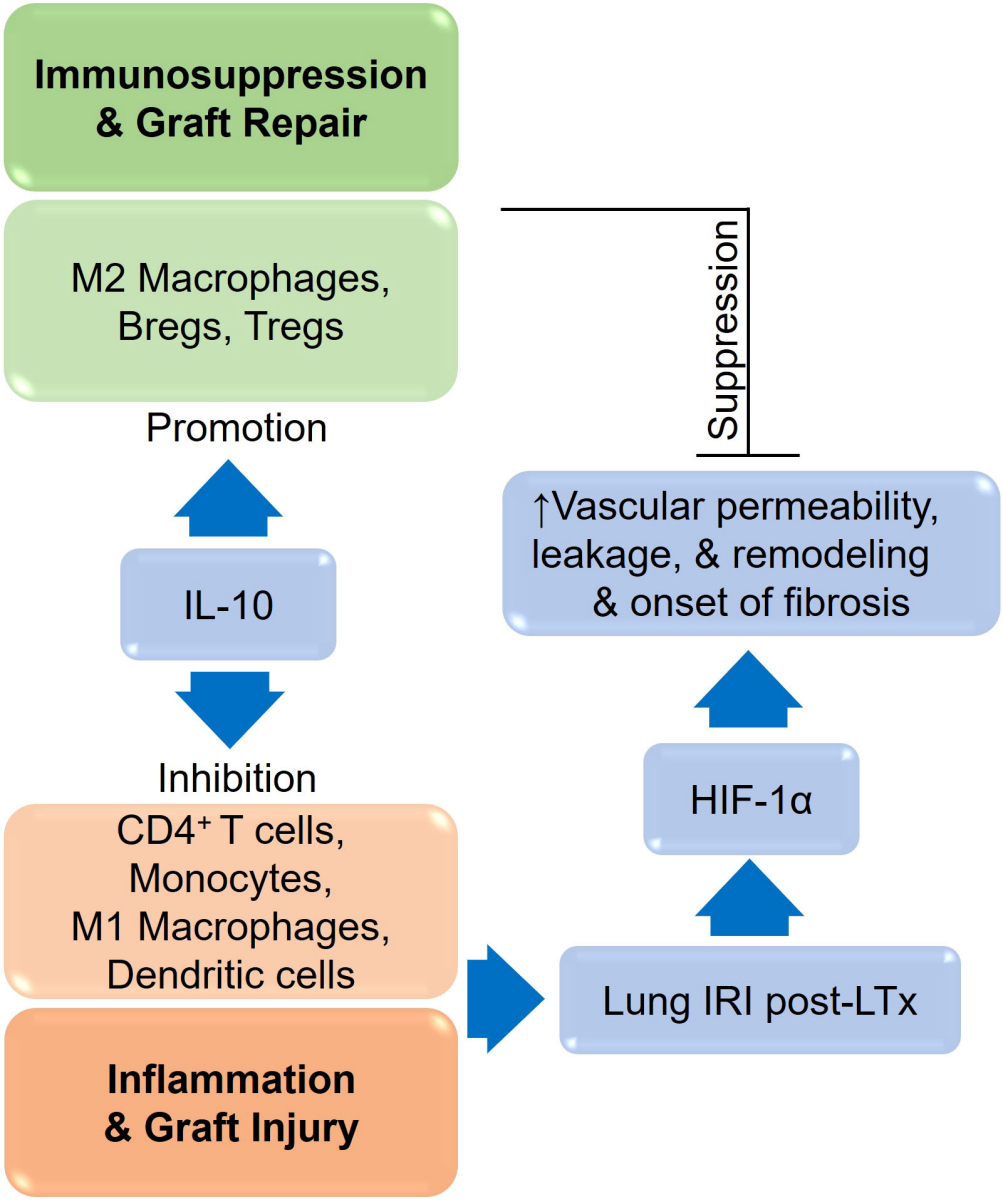
A schematic representation IL-10 and HIF-1α during tissue injury and repair.

In wound healing, several immune cells participate in the process, including platelets, neutrophils, macrophages, fibroblasts, lymphocytes, epithelial and endothelial cells. Wound healing is a complex process that goes through fibrotic remodeling, angiogenesis, cell migration and proliferation, and a discrete equilibrium of these stages to facilitate effective wound healing. This balance, however, is disrupted in chronic non-healing wounds, wherein the impaired microvascular blood supply and resulting ischemia cripple cellular functions and make it difficult to deliver the basic signaling molecules. However, Tregs, as well as their associated regulatory mediators, help to protect the tissue from inflammation (76, 117–120). During hemostasis, platelets release TGF-β1, PDGF, FGF-2, and VEGF to recruit neutrophils and macrophages, while neutrophils release ROS, NO, proteases, VEGF, and IL-17 to destroy pathogens (121–123). Besides, NK cells secrete IFN-γ, TNF-α and also release perforins and granzymes that are cytotoxic to infected cells (124). Moreover, neutrophils release TNF-α, IL-1β, IL-6, and MCP-1, which attract monocytes and dendritic cells and activate T cells that cause Th1 pro-inflammatory responses (125). In the inflammatory phase of acute wound healing, macrophages secrete IL-1, VEGF, FGF-2, TNF- α, IL-6, IFN-γ, TGF-β, and PDGF, which promote the proliferation of fibroblasts, keratinocytes, and epithelial cells, whereas in the remodeling phase IL-4, IL-10, and IL-13 induce the transition of M1 to M2 macrophages (122, 125). Besides, other cells, such as MSCs and fibroblasts, secrete TSG-6, which promotes wound healing by limiting macrophage activation, inflammation, and fibrosis (126, 127). M2 macrophages generally inhibit inflammation and promote tissue repair through IL-10 and TGF-β, which stimulate ECM synthesis, angiogenesis, and fibroblast proliferation (117). During inflammation, lymphocytes are also recruited to the wound and release IFN- γ, TGF-β, IL-10, IL-2, IL-17, and IL-22 (128). Later, angiogenesis replaces damaged vessels with granulation tissue, in which epidermal cells, fibroblasts, vascular endothelial cells, and macrophages produce β-FGF, TGF- β, and VEGF to bolster angiogenesis. VEGF induces angiogenesis through adenosine, which in turn stimulates hypoxia-induced proliferation, therefore A_2A_ receptors, is now considered a potent regulator of the early stages of wound healing (129, 130). Transitioning from a pro-inflammatory M1 macrophage-dominant wound to an anti-inflammatory M2 macrophage-dominant milieu is essential in resolving inflammation and preparing the wound for effective repair, which is further supported through the Tregs and associated mediators (63, 117, 120, 122, 131). IL-10, produced mainly by Tregs, monocytes, Th2 cells, subsets of activated T cells, and B cells, collectively restrains inflammation and immune responsiveness by regulating cell proliferation, and differentiation (132–134). IL-10 is mostly secreted by Tregs to combat inflammation, tissue repair, and antifibrotic events; however, IL-10 levels also regulate Treg expression of FOXP3 and suppressive activities (31, 70, 73, 74, 80, 132, 133, 135–146). Furthermore, Tregs promote tissue repair through various regulatory cytokines, which include IL-10, TGF-β, IL-33 and IL-35 (147, 148). In addition to limiting collateral tissue damage caused by uncontrolled immune responses, IL-10 helps maintain the regulatory microenvironment by upregulating TSG-6, M2 macrophages, and, tolerogenic dendritic cells (DC-10), antigen-specific T regulatory type 1 (Tr1), while suppressing Th1/Th17 effector immunity (60, 78, 80, 149–152). IL-10 is a potent antifibrotic, reparative, as well as vasculo-protective cytokine that assists in the repair of tissue following a sporadic alloimmune response during transplantation (60, 61, 76, 78, 80, 151, 153, 154). The anti-inflammatory properties of IL-10 help to suppress the production of pro-inflammatory cytokines such as IFN- γ, IL-2, IL-3, and TNF- α by Th1 cells, mast cells, NK cells, endothelial cells, eosinophils, and macrophages (79, 79, 132, 150, 155–158). Through the surface expression of TSG-6, FOXJ1, Fascin-1, and β-catenin proteins, IL-10 enhances microvascular supply, tissue oxygenation, and airway epithelium regeneration in allografts, further supporting the therapeutic benefits during wound healing and tissue repair (60, 61, 76, 159) (**Figure 5**).

## Conclusion

We conclude that a molecular pathway and associated alloimmune response and microvascular disruption during airway anastomosis play a crucial role in the development of airway complication as replicated in a mouse model of orthotopic tracheal transplantation model, which replicates airway complications as seen in clinical conditions. Understanding these molecular pathways aids in developing targeted therapeutic strategies, such as antioxidant therapies, angiogenic growth factors, and immunomodulation, to improve anastomotic healing and reduce complications.
